# The association between disordered eating and health-related quality of life among children and adolescents: A systematic review of population-based studies

**DOI:** 10.1371/journal.pone.0222777

**Published:** 2019-10-04

**Authors:** Xiu Yun Wu, Wen Qiang Yin, Hong Wei Sun, Shu Xiang Yang, Xin Yang Li, Hong Qing Liu

**Affiliations:** School of Public Health and Management, Weifang Medical University, Weifang, Shandong, China; King's College London, UNITED KINGDOM

## Abstract

**Background:**

Previous studies have documented that disordered eating is associated with a wide range of impaired physical and mental health conditions among children and adolescents. The relationship between disordered eating and health-related quality of life (HRQOL) has been predominantly examined in children and adolescents who are overweight or obese or suffer from chronic illnesses. In the last decade, several studies have been conducted to investigate the relationship between disordered eating and HRQOL among school and community children and adolescents. No systematic review or meta-analysis has synthesized the findings from these population-based studies. The purpose of this systematic review and meta-analysis was to synthesize the relationship between disordered eating and HRQOL among the general population of children and adolescents.

**Methods:**

We performed a computer search for the English language literature using the databases PUBMED, EMBASE and PSYCINFO to retrieve eligible studies published between 1946 and August 9, 2018. We also searched the relevant articles using PubMed related article search features and manually examined the reference lists of the retrieved full text articles selected from the database search. The association between disordered eating and HRQOL was synthesized using both a qualitative method and a meta-analysis. The review was conducted adhering to the Preferred Reporting Items for Systematic reviews and Meta-Analyses (PRISMA) guidelines.

**Results:**

We identified eight studies that met the inclusion criteria and were included in the final synthesis. The studies included six cross-sectional studies and two longitudinal studies. The systematic review found that disordered eating attitudes and behaviors were associated with lower HRQOL among children and adolescents. Children and adolescents with bulimia nervosa (BN), binge eating disorder (BED), purging disorder (PD) and other eating disorder symptoms had poorer HRQOL than their healthy peers without the eating disorder conditions. The meta-analysis using four out of the eight studies showed that disordered eating was significantly associated with poor psychosocial health and lower overall HRQOL among children and adolescents.

**Conclusion:**

The present review reveals that disordered eating behaviors and eating disorders are associated with decreased HRQOL in children and adolescents. More prospective studies are needed to ascertain the directions in the relationship between disordered eating and HRQOL among children and adolescents. The findings of this review suggest that health programs for promoting healthy eating and reducing disordered eating behaviors among school children and adolescents may help to enhance the HRQOL and overall health status of these individuals.

## Introduction

Disordered eating encompasses a wide range of abnormal eating behaviors with different levels of severity [1]. Although the prevalence of clinically diagnosed eating disorders appears low, disordered eating symptoms and behaviors, such as fasting, food intake restriction, vomiting, binge eating and purging behaviors, are very common in children and adolescents [1,2]. The disordered eating behavior that does not fully meet the criteria for the diagnosis of an eating disorder can be considered a subclinical form of eating disorder [3]. These disordered eating symptoms and behaviors are often screened and measured using self-reported scales [1,3]. Eating disorders (EDs), including anorexia nervosa (AN), bulimia nervosa (BN) and binge eating disorder (BED), are regarded as more severe types of disordered eating [1,2]. AN is defined by persistent restriction of nutrient intake and an intense fear of gaining weight, leading to an abnormal underweight status [3,4]. BN is characterized by recurrent episodes of binge eating, followed by compensatory behavior, such as vomiting and fasting [4,5]. BED is defined by repeated episodes of binge eating accompanied by a sense of loss of control [4,5].

Disordered eating among children and youth is a public health challenge because it is a risk factor for poor physical and mental health consequences in children and adolescents. Previous studies have documented that disordered eating behaviors are associated with various adverse physical health consequences, such as obesity, malnutrition and diabetes, among children and youth [6–8]. There is also evidence that disordered eating behaviors are associated with impaired psychosocial and mental health consequences, including depression, anxiety, psychological stress, low self-esteem, and suicidal ideation among children and adolescents [9–13]. Disordered eating during childhood and adolescence is associated with an increased risk for both physical and mental health disorders later in life [9,14–16].

Health-related quality of life (HRQOL) is a subjective evaluation of the overall health of an individual as well as the health of underlying subdimensions of physical, psychological and social functioning and well-being [17]. Research has shown that healthy dietary behavior and good diet quality are associated with better HRQOL among children and adolescents [18–21]. Regarding the effect of disordered eating behavior on the HRQOL of children and adolescents, prior research has been predominantly carried out among children and adolescents with obese or disease conditions (e.g., diabetes) or with extreme severe eating disorders in clinical settings [22–25]. In recent years, we have witnessed several population-based studies that examined the relationship between disordered eating and HRQOL using community or school samples of children and adolescents [1,2,26]. However, we have not found a systematic review study for the relationship between disordered eating behaviors and HRQOL among children and adolescents. Investigations of the relationship between disordered eating and HRQOL among the population of children and adolescents will help provide an evidence base to inform the develop of population-based intervention programs to promote healthy eating. Particularly, if disordered eating could lead to impaired QOL in child and youth populations, then the intervention programs tailored toward preventing disordered eating and eating disorders among the young populations would be beneficial to the improvement of their health status.

In a systematic review of 12 cross-sectional and 5 cohort studies, we recently reported that diet quality and dietary behaviors, in terms of diet habits or patterns (e.g., fast foods, breakfast skipping, Mediterranean diet, fruits and vegetables), were associated with HRQOL in children and adolescents and the exposure-outcome relationship expressed in a dose-response manner [18]. The present systematic review extended the previous research by specifically exploring and synthesizing the relationship between disordered eating behaviors, including diagnosed eating disorders and HRQOL among the children and adolescents. These disordered eating behaviors (e.g., AN, BN) as described above are distinct from general habitual dietary intakes or patterns [27] and are considered more severe episodes of chronic disordered eating or mental disorder illnesses. Thus, it is worthwhile to investigate the relationship of these disordered eating behaviors and symptoms with the health outcome of HRQOL among children and adolescents.

The present systematic review aimed to synthesize how disordered eating behaviors influence HRQOL among the population of school and community children and adolescents. We hypothesized that children and adolescents who suffered from a disordered eating behavior or an eating disorder would have a lower HRQOL than their peers who did not have a disordered eating behavior or an eating disorder.

## Methods

### Literature search

We performed a computer search for English language literature in PUBMED (1946 to July 18, 2018), EMBASE (1966 to June 19, 2018), and PSYCINFO (1980 to August 9, 2018). MeSH headings and keywords included ‘eating disorder’, ‘disordered eating’, ‘dietary or eating behavior’, ‘binge eating’ ‘anorexia nervosa’, ‘bulimia nervosa’, ‘bulimic eating disorder’, ‘quality of life’, ‘health status’, ‘children’, ‘adolescents’, ‘boys’, ‘girls’, ‘teen’, ‘youth’. A general search strategy with a combination of the above search terms was used: (eating disorder* OR disordered eating OR binge eating OR anorexia nervosa OR bulimic eating disorder OR bulimia nervosa OR dietary behav* OR eating behav*) AND (child* OR adolescen* OR teen* OR boys OR girls OR youth) AND (health status OR quality of life). We also searched the literature using PubMed ‘related article’ searching and manually examined the reference lists of the extracted included studies and systematic reviews to retrieve other eligible studies. The full search terms and the search strategy used in each of the databases are presented in S1 Table in the supporting information file.

### Inclusion and exclusion criteria

We followed the PICO approach [28] in the specification of the study inclusion and exclusion criteria: Population (P), Intervention or Exposure (I or E), Comparison (C), Outcome (O). Studies were selected based on the following inclusion criteria: the study population included children and adolescents aged between 3 years and 19 years, defined as children and adolescents in communities or schools in a geographic region or a country (P). These children and adolescents generally have different demographic characteristics and health status (e.g., type and severity of diseases) compared to their peers in clinical settings (e.g., patients in health care institutions) or in intervention programs [22–25]. In the case of longitudinal studies with a follow-up age greater than 19 years, the age at follow-up was not restricted. The exposure included disordered eating attitudes and behaviors, eating disorders, binge eating, anorexia nervosa, bulimia nervosa and bulimic-type eating disorders (E). The study design included cross-sectional, cohort and case-control studies that aimed to evaluate the relationship of disordered eating exposure with HRQOL (C). The primary outcome of the study was quality of life (QOL) or health-related quality of life measured by a multidimensional HRQOL instrument (O).

The exclusion criteria included studies that examined the association between disordered eating or eating disorder and HRQOL in children and adolescents using clinical samples or among adults or in overweight/obese children and adolescents; studies that used a single item of self-rated or self-perceived health status as an outcome; reviews, meta-analyses, clinical trials, comments, letters, case reports and guidelines.

### Study selection

The electronic database search for the studies was conducted by the first author (XYW), and the obtained titles and abstracts of the studies were then screened and evaluated for selection using the predefined inclusion and exclusion criteria. The full text articles of the potential eligible studies were retrieved for detailed examination. Studies for which the first author was uncertain about the eligibility, the full text articles were then reviewed for eligibility by other reviewers (SHXY, XYL). Finally, the reference lists of the included studies and systematic reviews identified from the electronic database search were comprehensively examined to retrieve additional eligible studies. Disagreements on the eligibility of the selected studies were resolved by discussion among all researchers.

### Data extraction and data synthesis

A data extraction form was used to extract data from each of the included studies. Data for the qualitative synthesis included the following information: first author of the study, publication year, country of the study, study design, sample characteristics (e.g., sample size, mean age and age range of the participants), measures used to assess disordered eating and measures used to assess HRQOL, key data analytical methods, main findings and risk of bias assessment for each study.

For quantitative synthesis, we performed a meta-analysis for the studies with HRQOL data of similar scales to estimate the pooled association between disordered eating behaviors or attitudes and HRQOL. To account for heterogeneity across the studies, we used a random effects model. The I^2^ statistic and Q test were used to test the degree and statistical significance of heterogeneity, respectively. An I^2^ >50% and p<0.1 indicate substantial and significant heterogeneity between studies [29]. The pooled estimates were reported as the standardized mean difference in the HRQOL scores with their 95% confidence intervals across the studies. The meta-analysis was performed using statistical software from Review Manager 5.2 (The Cochrane Collaboration, Copenhagen Denmark).

We reported the review in accordance with the Preferred Reporting Items for Systematic reviews and Meta-Analyses (PRISMA) statement [28].

### Assessment of the risk of bias

The Newcastle-Ottawa Scale was used to assess the risk of bias of the included studies [30]. The scale contains eight items covering three components: selection, comparability and outcome. Each item can be scored as one point or two points and summed up to a total score ranging from 0 (highest risk of bias) to 9 (lowest risk of bias). The study quality was then categorized into three groups based on the risk of bias: high risk of bias (0–4), moderate risk of bias (5–6), and low risk of bias (7–9) [31,32].

## Results

### Characteristics of the included studies

**Fig 1:**
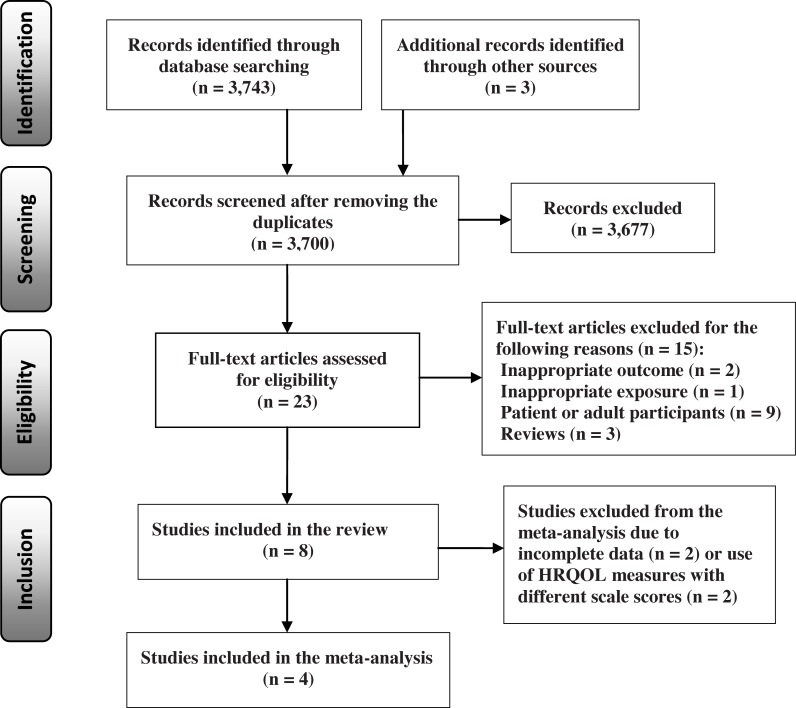
PRISMA flow diagram for the selection of the included studies.

The electronic database search identified 3,743 relevant published papers for eligibility screening, including PUBMED (n = 1,950), EMBASE (n = 691), and PSYCINFO (n = 1,102). An additional three studies were identified by searching PubMed for related articles and the reference lists of the existing relevant studies. After the title and the abstract review and exclusion of the duplicate references (n = 46) between the databases, 23 full text articles were retrieved for a detailed examination for eligibility using the predefined inclusion and exclusion criteria. Of these studies, 15 were further excluded due to patient samples (e.g., with overweight or obesity) or adult participants (e.g., college students), inappropriate outcomes or exposures, and reviews [5,11,13,22–24,33–41]. As a result, a total of eight studies were included in the final synthesis in the current review [1,2,26,42–46]. The procedure for the selection of the studies is presented in the PRISMA flow diagram (Fig 1).

Table 1 shows the sample characteristics of the included studies, the assessment of disordered eating behaviors or attitudes, and the measurement of HRQOL. The included studies were published between 2008 and 2017. The studies were conducted in six countries, including Australia (n = 2), the United States (n = 2), Germany (n = 1), Austria (n = 1), Greece (n = 1), and Iran (n = 1). Most studies were cross-sectional (n = 6). Two studies used a longitudinal design. The sample size of the individual studies was between 130 (smallest) [26] and 3,610 (largest) [2], with a total of 9,666 participants for all included studies.

**Table 1:** Characteristics of the included studies, assessments of disordered eating and HRQOL.

Author, publication year and country	Study design, samples	Assessment of disordered eating	Assessment of HRQOL
1. Mitchell et al., 2017 USA [26]	Longitudinal study, one-year follow-up, three time points: time 1 (baseline), time 2 (6-months), time 3 (12 months). Children, n = 130, 46% boys, Age range: 7–10 at baseline. Mean age: 8.62 (SD1.11).	Self-report Disordered eating attitudes and behaviors were measured at all 3 time points by the Children’s Eating Attitudes Test (ChEAT), with 20 items on a six-level Likert scale from ‘never’ to ‘always’. The total score was used for analyses in the study.	Self-report The Pediatric Quality of Life Inventory (PedsQL 4.0), four subscales (physical, emotional, social, and academic functioning), two summary scores (physical and psychosocial QOL), and total score, assessed at all 3 time points.
2. Zervaki et al., 2017 Greece [42]	Cross-sectional study Adolescents, n = 400, 50.5% girls, Age range: 14–17.	Self-report Disordered eating attitudes were assessed by the Eating Attitudes Test-26 (EAT-26), three subscales: Dieting scale, Bulimia and Food Preoccupation scale and Oral Control scale. A total score at or above 20 in the EAT-26 indicates abnormal eating behavior.	Self-report The KIDSCREEN-27. 27 items, five dimensions: physical well-being, psychological well-being, autonomy and parents’ relation, social support and peers, and school environment. The scale scores were transformed into T-values with mean (±SD) scores of 50 ±10. A higher score indicates higher HRQOL.
3. Mitchell et al., 2016 USA [43]	Cross-sectional study Children, n = 165, 50% boys, Age range: 8–12. Mean age: 9.41 (SD1.01).	Self-report The Children’s Eating Attitudes Test (ChEAT) was used to assess disordered eating attitudes and behaviors, with 20-items, 6-point Likert scale (never, rarely, sometimes, often, very often, always). The total score (excluding 3 questions on social pressure to gain weight subscale) was used in the study.	Self-report The PedsQL 4.0. Total score for the PedsQL was used in the analysis.
4. Zeiler et al., 2016 Austria [2]	Cross-sectional study Adolescents, n = 3,610, 44.7% boys, Age range: 10–18. Mean age: 11.02 (SD0.55) for fifth graders, 12.99 (SD0.56) for 7th graders, 15.08 (SD0.69) for 9th graders, 17.33 (SD1.04) for 11th graders.	Self-report The SCOFF questionnaire, five items for screening eating disorders (EDs), including intentional vomiting, loss of control of overeating, weight loss, body dissatisfaction and food intrusive thoughts; item responses: ‘yes (score = 1)’ versus ‘no (score = 0)’. A SCOFF score ≥2 represents having a risk for an ED.	Self-report The KIDSCREEN scales: KIDSCREEN-10, self-perception, parent relation and home life, social support and peers, school environment and bullying. Items are rated on a 5-point scale. Raw scores are transferred into T-scores based on German normative data.
5. Jalali-Farahani et al., 2015 Iran [1]	Cross-sectional study Adolescents, n = 465, 51.2% boys, Age range: 14–17. Mean age: 15.55 (SD0.94).	Self-report The Eating Attitudes Test-26 (EAT-26): 26 items with three subscales: Dieting, Bulimia and food preoccupation, Oral control. Each item was on a six-point Likert scale ranging from a response of ‘always’ to ‘never’. The item (except for item 26) response was scored as zero for ‘Sometimes’, ‘Rarely’ and ‘Never’, one for ‘Often’, two for ‘Usually’ and three for ‘Always’. Item 26 is scored reversely. A total score of 20 and above was classified as at-risk of eating disorders (disordered eating).	Self-report The PedsQL 4.0. A higher score indicates a better HRQOL.
6. Bentley et al., 2015 Australia [44]	Cross-sectional study Adolescents, n = 1,670, 32% boys, Age range: 12–18. Mean age: 14.85 (SD1.70) for males, 15.51 (SD1.63) for females.	Self-report The Eating Disorder Examination Questionnaire (EDE-Q): 36-item measure that assesses eating disorder pathology during the past 28 days. 22 items of the EDE-Q constitute four subscales: Restraint, Eating concerns, Weight concerns and Shape concerns. A global score indicates overall level of eating disorder pathology was derived from the 22 items. The remaining 14 items of the EDE-Q assess the key eating disorder behaviors: objective binge eating, the use of purging and excessive exercise as the means of controlling weight or shape. One additional item was included to assess the occurrence and frequency of subjective binge eating episodes.	Self-report The Pediatric Quality of Life Inventory^TM^ 4.0–Short Form (PedsQL^TM^ 4.0 SF15), 15-items, four domains: Emotional wellbeing (4 items); Social functioning (3 items); Academic functioning (3 items); and Physical health (5 items). Responses are on a five-point Likert scale ranging from ‘never’ to ‘almost always’. Scores are transformed to a standard scale ranging from 0 to 100, with higher scores indicating higher QOL. The Psychosocial Health Summary score (the average of scores on the Emotional wellbeing, Social functioning and Academic functioning domains) was used in the study.
7. Allen et al., 2013 Australia [45]	Cohort study, followed from 14 to 20 years old, and Cross-sectional study. Adolescents, n = 1,383, 49% males, Age range: 13.00–15.08 years, Mean age: 14.01 (SD0.19) at baseline; Age range: 19.00–22.08, Mean age: 20.01 (SD0.44) at last follow-up.	Self-report Eating disorder symptoms were assessed using 24 items adapted from the Child Eating Disorder Examination (ChEDE) and the Eating Disorder Examination-Questionnaire (EDE-Q). Four response options were used for all items: 0 = not at all; 1 = some of the time (once per week/a few times a month); 2 = a lot of the time (a few times a week); and 3 = most of the time (every day or nearly every day).	Self-report The 12-item Short-Form Health Survey-12 (SF-12) was used to assess physical and mental QOL at age 20 (not collected at age 14 or 17). The norm-based score with a population mean of 50 (SD = 10) was used in the study.
8. Herpertz-Dahlmann et al., 2008 Germany [46]	Cross-sectional study Adolescents, n = 1,843, 51.3% boys (n_boys_ = 945), Age range: 11–17 years at baseline. Mean age: 14.6 (SD2.0).	Self-report Disordered eating behaviors (anorexia or bulimia nervosa, atypical eating disorders) were assessed using the SCOFF instrument with five questions: deliberate vomiting, loss of control of overeating, weight loss, body image distortion and the impact of food on life. Two positive answers in the five questions indicate a presence of disordered eating behavior and attitudes.	Self-report and parent-report The generic KINDL-R Questionnaire, 24 Likert-scaled items covering six domains: physical well-being, psychological well-being, self-esteem, family, friends, and everyday functioning (school). The score was transformed to range between 0 and 100 with higher values indicating better HRQOL.

**Abbreviations:** HRQOL: health-related quality of life; PedsQL: Pediatric Quality of Life Inventory; SF-12: Short-Form Health Survey-12; SD: standard deviation

Regarding the assessment of the disordered eating attitude and behavior, two studies used ‘the Children’s Eating Attitudes Test (ChEAT, 20 items)’ [26,43], two studies applied ‘the Eating Attitudes Test-26 (EAT-26)’ [1,42], and two studies utilized ‘the SCOFF instrument (five items)’ [2,46]. The remaining two Australian studies used ‘the Eating Disorder Examination Questionnaire (EDE-Q)’ and ‘the Child Eating Disorder Examination (ChEDE)’, respectively.

Half of the included studies (n = 4) assessed HRQOL using the Pediatric Quality of Life Inventory version 4.0 questionnaire (PedsQL 4.0) [1,26,43,44]. Other HRQOL measures that were used in the other studies included the KIDSCREEN-27, the KIDSCREEN-10, the 12-item Short-Form Health Survey-12 (SF-12) and the KINDL-R questionnaire.

### Risk of bias assessment

Table 2 presents the key finding for the association between disordered eating and health-related quality of life and the risk of bias score for each study. In general, the study quality of the included studies was low to moderate. Seven studies were rated at a moderate risk of bias (score: 5–6), and one study at high risk of bias (score = 4). The low quality was mainly due to the cross-sectional design, small sample size, and inadequate statistical analysis (e.g., lack of control for potential confounders).

**Table 2:** The main findings, statistical methods and the risk of bias score of the included studies.

Author, publication year	Summary of the main findings	Key statistical methods and covariates	Risk of bias score
1. Mitchell et al., 2017 [26]	• Disordered eating was negatively correlated with both physical and psychosocial HRQOL (PedsQL 4.0) at Time 1 and Time 2 (p<0.01); disordered eating at Time 1 and Time 2 was only negatively associated with HRQOL at Time 3 (p<0.01). • Path analysis indicated that disordered eating attitudes and behaviors did not significantly predict physical HRQOL across any time point. Higher disordered eating attitude and behavior scores at Time 2 were associated with subsequent lower psychosocial HRQOL scores at Time 3 (β = -0.33, SE = 0.12, p = 0.01).	**Statistical methods:** Correlation analysis, path regression model. **Covariates:** Child’s sex, grade level, race/ethnicity, and BMI.	6
2. Zervaki et al., 2017 [42]	• 16.6% of the total sample of students (13.7% of boys and 19.3% of girls) reported having disordered eating attitudes. • Disordered eating attitudes were negatively correlated with the total score of HRQOL as measured by the KIDSCREEN-27 (correlation coefficient (CC) = -0.142, p<0.01) and with the subscales: Physical well-being (CC = -0.115, p<0.01), parents and autonomy (CC = -0.137, p<0.01) and school environment (CC = -0.178, p<0.001).	**Statistical methods:** Correlation analysis. **Covariates:** Not applicable.	4
3. Mitchell et al., 2016 [43]	Mean total PedsQL score = 76.92, SD = 14.54. Disordered eating attitudes and behaviors were negatively associated with HRQOL (β = -5.99, SE = 1.92, p<0.001 for direct effect of the exposure on the outcome in the mediation model).	**Statistical methods:** Pearson correlations, path (mediation) model. **Covariates:** Child’s age, sex and BMI.	5
4. Zeiler et al., 2016 [2]	• 23.55% [95% confidence interval (CI): 22.2–25.9] of adolescents had a risk for eating disorders. • Eating disorder (SCOFF total score≥2) was significantly associated with reduced HRQOL (KIDSCREEN-10 total score: β = -0.20, p<0.001 in hierarchical linear regression). SCOFF scores ≥2 were associated with worse QOL in all the KIDSCREEN-10 dimensions: the school environment, self-perception, bullying, the social support and peer relationships. All SCOFF items except for weight loss were independent predictors for HRQOL.	**Statistical methods:** Analyses of covariance, hierarchical linear regression. **Covariates:** Sex, age, BMI, and general psychopathology.	6
5. Jalali-Farahani et al., 2015 [1]	The total and all subscale scores of HRQOL (PedsQL 4.0) were significantly lower in adolescents with disordered eating compared to those adolescents without the condition (t value ranges from 2.75 to 5.21, p<0.05). The mean total PedsQL score (±SD) was 81.56 ±11.21 for adolescents without disordered eating, and 74.70 ±13.96 for the peers with disordered eating. Adolescent girls who had disordered eating had poorer emotional functioning and social functioning of HRQOL, and total HRQOL score compared with those who did not have disordered eating (t = 2.05, t = 2.31, t = 2.03 respectively, p<0.05). Emotional functioning and school functioning of HRQOL, and total HRQOL score were poorer among adolescent boys who had disordered eating compared to those who did not have the disorder (t = 3.68, 3.08 and 3.37 respectively, p<0.05).	**Statistical methods:** Independent samples T-test, simple linear regression. **Covariates:** Not report.	5
6. Bentley et al., 2015 [44]	• Small to moderate correlations were observed between the occurrence of eating disorder features (EDF) and impairment in HRQOL for both male and female adolescents (correlation coefficient: -0.09 to -0.38). • ANCOVA indicated that adolescents who reported EDFs (extreme dietary restriction, objective binge eating, purging, excessive exercise, and weight/shape overvaluation) had higher levels of Psychological distress and poorer QOL (Psychosocial Health Summary score) than those who did not report EDF (all p<0.01). • Subjective binge eating was associated with elevated levels of distress in girls (t = -11.74, p<0.05) but not in boys (t = -2.11, p = 0.59), and with greater impairment in QOL in girls (t = 11.07, p<0.01) than in boys (t = 2.07, p<0.05).	**Statistical methods:** Pearson correlation analysis, Independent sample T-test, Analysis of covariance (ANCOVA). **Covariates:** Demographic variables (other than sex) in ANCOVA.	5
7. Allen et al., 2013 [45]	• At age 20 (follow-up), physical health QOL (the SF-12) did not differ significantly by eating disorder category, but mental health QOL was significantly lower in males with a DSM-IV-TR eating disorder and with a DSM-5 eating disorder than those peers with no eating disorders. • At age 20 (follow-up), bulimia nervosa (BN), binge eating disorder (BED) and purging disorder (PD) were associated with lower mental health QOL in females, but not physical health QOL.	**Statistical methods:** Nonparametric Kruskal-Wallis and Mann-Whitney U tests. **Covariates:** Not applicable.	6
8. Herpertz-Dahlmann et al., 2008 [46]	Adolescents with a disordered eating behavior had significantly lower QOL for all of the KINDL-R-subscales and for the total score (effect size = 0.081, considered as moderate effect size).	**Statistical methods:** Analysis of covariance. **Covariates:** Age, sex, BMI.	5

### The association between disordered eating and HRQOL

#### Findings of the qualitative data synthesis

All included studies observed a significant association between disordered eating and HRQOL among children and adolescents (Table 2). Mitchell et al. (2017) found that higher disordered eating attitude and behavior scores at 6 months (Time 2) were associated with lower psychosocial HRQOL scores at 12 months (Time 3) (β coefficient = -0.33, standard error (SE) = 0.12, p = 0.01 in the path regression model) using the PedsQL 4.0 after controlling for variables, such as sex, grade, race/ethnicity and body mass index [26]. In another study by Mitchell et al. (2016), disordered eating attitudes and behaviors were found to be associated with a decreased total HRQOL score (β coefficient = -5.99, SE = 1.92, p<0.001) [43]. Zeiler et al. (2016) observed in a sample of 3,610 participants that adolescents who experienced an eating disorder (the SCOFF total score≥2) had significantly reduced HRQOL, as measured by the KIDSCREEN-10 total score (β coefficient = -0.20, p<0.001) [2]. The regression model was adjusted for sex, age, BMI and general psychopathology. This study also observed that eating disorder was associated with worse HRQOL in the following domains: school environment, self-perception, bullying, social support and peer relationships [2]. Jalali-Farahani and colleagues reported that adolescent girls who reported having disordered eating had poorer emotional and social functioning and lower overall HRQOL relative to healthy peers [1]. Disordered eating was related to poorer emotional functioning, school functioning and total HRQOL among adolescent boys. Australian studies reported that adolescents who had eating disorders experienced lower quality of life [44,45]. Allen et al. also observed that eating disorder was associated with lower mental QOL but not physical QOL [45]. Herpertz-Dahlmann et al. found that adolescents with disordered eating behavior had significantly decreased HRQOL for all KINDL-R subscales and for the total HRQOL score [46]. Zervaki et al. (2017) observed that disordered eating attitudes correlated with a decreased total HRQOL as measured by the KIDSCREEN-27 (correlation coefficient (CC) = -0.142, p<0.01) and a decreased QOL in the following subscales: physical well-being (CC = -0.115, p<0.01), parents and autonomy (CC = -0.137, p<0.01) and school environment (CC = -0.178, p<0.001) [42].

#### Findings of the meta-analysis

**Fig 2:**
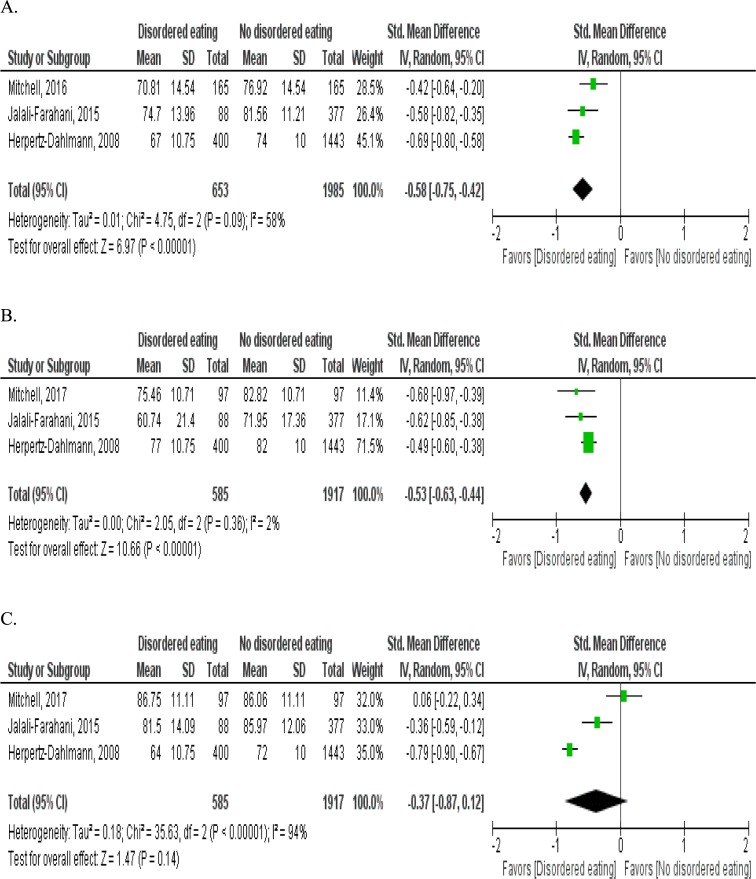
**Forest plot of mean differences in total (A), psychosocial (B) and physical (C) domains of HRQOL between the disordered eating group and the non-disordered eating group HRQOL measures:** The PedsQL 4.0 in the studies by Mitchell and Jalali-Farahani, 2015; The KINDL-R in the study by Herpertz-Dahlmann, 2008 **Disordered eating variables:** The Mitchell study used the total score of six-point Likert scale variable of disordered eating, the mean difference represents the decrease in QOL for one unit increase in the disordered eating; Jalali-Farahani, 2015 and Herpertz-Dahlmann, 2008 used dichotomous variable of disordered eating (with and without disordered eating behavior). Std: standardized; SD: standard deviation; CI: confidence interval.

Four studies were included in the meta-analysis [1,26,43,46]. The remaining four studies were excluded from the meta-analysis due to non-availability of HRQOL data or due to the use of a measure of HRQOL with different scale scores [2,42,44,45]. In the meta-analysis, a significant difference in the mean total HRQOL score was observed between children and adolescents with disordered eating and those without the disordered eating condition (pooled standardized mean difference (SMD) = -0.58, 95% CI: -0.75, -0.42, p<0.0001) for three studies (Fig 2A). Disordered eating was significantly associated with poor psychosocial health (pooled SMD = -0.53, 95% CI: -0.63, -0.44, p<0.0001) (Fig 2B). The association of disordered eating with physical QOL was not significant (pooled SMD = -0.37, 95% CI: -0.87, 0.12, p = 0.14), with high between study heterogeneity (I^2^ = 94%, p<0.001). The high heterogeneity across studies for the relationship between disordered eating and physical QOL may be due to the differences between the studies in the measurement of the disordered eating behavior and the HRQOL (Fig 2C).

## Discussion

In the present review, we found that disordered eating behaviors or attitudes were associated with impaired HRQOL in children and adolescents. The associations were observed for both total HRQOL and specific domains including physical functioning, school functioning, emotional functioning, psychosocial quality of life, school environment, social support and peer relationships.

The relationship between disordered eating and HRQOL has been comprehensively investigated in adult populations [10,36,39,47–50] and in children and youth with disease conditions [22,25]. Some studies have intensively examined the association between disordered eating attitudes and behaviors and HRQOL in overweight and obese children and adolescents [23,24,37,41]. These studies showed that overweight and obese children and adolescents who engaged in disordered eating behaviors (e.g., AN, BED, ED symptoms) experienced serious impairments in HRQOL, and the impaired effect appeared greater in the mental health component of HRQOL than in the physical health component. However, as children and adolescents with overweight and obese status or chronic diseases tend to have more severe disordered eating behaviors and eating disorder illnesses relative to the peers in school or community settings, these findings may not be generalizable to children and adolescents in communities or schools. To our knowledge, the present review is the first to investigate school- or community-based studies to synthesize the relationship between disordered eating and HRQOL among children and adolescents. The findings of the study are in line with previous studies among adults and with studies using clinical samples of children and youth, showing that people with disordered eating had significantly poorer HRQOL [36,40,41,47,48,50].

When the effect of disordered eating on specific domains of HRQOL is considered, the present review observed that disordered eating was associated with multiple dimensions of HRQOL, including both physical and mental dimensions of HRQOL. Five studies found a significant correlation between disordered eating and physical functioning or physical well-being of HRQOL [1,2,26,42,46]. Several primary studies have also documented that eating disorder was associated with greater impairments on psychosocial QOL and emotional functioning than on physical QOL [1,26,45]. Other HRQOL dimensions that were related to disordered eating included school functioning or environment, social support, and relationships with parents and peers [1,2,42,46]. This observation is consistent with other studies in overweight/obese children and youth and young adults [23,24,39]. Additionally, several studies in the present review analyzed the effect of disordered eating on HRQOL with adjustment for the effect of demographic variables (e.g., age, gender) and BMI [2,26,43,46]. Hence, this finding suggests that disordered eating behavior may be an independent risk factor for HRQOL among children and adolescents, regardless of their body weight status, age and gender.

Currently, the study of a prospective or long-term relationship between disordered eating and HRQOL in children and youth is sparse. Two studies in our review used a longitudinal study design [26,45]. However, only one of these studies analyzed a prospective association between disordered eating at a previous time point and HRQOL at a follow-up time [26]. The analysis in the study by Allen et al. used the follow-up data for both exposure and outcome [45] and thus essentially revealed a cross-sectional association between disordered eating and HRQOL. Future research is needed to expand longitudinal studies to better understand the prospective or temporal relationship between disordered eating and HRQOL among the population of children and youth.

The data synthesis revealed that the magnitude of difference in HRQOL between children and youth with and without the disordered eating conditions exceeded the minimally important difference (MID) value for some common HRQOL measures. The MID represents a meaningful difference in a health outcome value that was perceived, on average, as beneficial by the patients and was considered as having clinical importance for altering treatments or health intervention strategies to improve the health status of patients or general populations [51]. The MID value varies with HRQOL measures. For example, a 4.5-point difference across comparison groups was considered as the MID value for the PedsQL measure [52]. The results in the current study showed that the overall differences of HRQOL between the disordered eating groups exceeded the MID value of 4.5 points for the PedsQL measure [1,26,43], suggesting that this difference may have some clinical or practical importance.

In the present review, the synthesis of the results was mainly based on a qualitative synthesis (Table 2). Due to the limited data available for the meta-analysis, we performed meta-analyses for four studies (Fig 2). We applied a random effect model to account for the heterogeneity across studies and reported the standardized mean difference to improve comparability in the outcomes between studies. However, the number of studies in the meta-analysis was small; thus, the statistical power may be limited in the estimation of the overall effect, as demonstrated in previous studies [53]. The result based on three studies in the meta-analysis showing that disordered eating was significantly associated with poor psychosocial health and total HRQOL is consistent with the finding of the qualitative synthesis. Although the meta-analysis did not observe a significant overall association of disordered eating with the physical dimension of QOL, the qualitative data synthesis based on the finding from five studies [1,2,26,42,46] found a significant relationship between disordered eating and physical HRQOL. The meta-analysis result could be partly attributed to the observation of no significant change of the physical QOL score over a six-month period in the study by Mitchell et al. (2017). Because only two HRQOL measures (the PedsQL 4.0 and the KINDL-R) were evaluated in the meta-analysis, this finding is limited to being representative of other outcome measures of HRQOL. To facilitate the quantitative synthesis using meta-analysis, future research is recommended to increase studies that use similar measures of HRQOL to those measures in this study and to extend relevant studies in different countries.

### Implications for research and population health interventions

Disordered eating behaviors are regarded as a public health concern among children and adolescents [2,54]. Although the prevalence of disordered eating behaviors and diagnosed eating disorders documented in the literature varies depending on the definition of disordered eating behaviors, it remains high in the population of children and adolescents using a broad definition of disordered eating behaviors [2,54]. A US population-based study among adolescents reported that 56.1% of girls and 29.1% of boys had disordered eating behaviors [54]. High prevalence figures were also observed in European countries [2,9,55]. Moreover, the high prevalence of disordered eating behaviors in adolescents tends to be stable and increases from adolescence to early adulthood [54–56]. The high prevalence of disordered eating behaviors and their harmful consequences on the health status of children and adolescents observed in the present review and in other studies highlight the pressing need for effective prevention programs targeting disordered eating behaviors and the promoting healthy behaviors among children and adolescents. Given that most of the included studies were cross-sectional, in future research, more longitudinal or prospective studies are needed to strengthen the level of evidence for a temporal and a causal relationship between disordered eating and HRQOL. Additionally, further research is needed to clarify the differential effect of different types of disordered eating behaviors on HRQOL among children and adolescents.

### Strengths and limitations

Strengths of the review include a comprehensive literature search for the eligible studies, the inclusion of multidimensional HRQOL measures allowing for the analysis of the effect of the exposure on overall and domain-specific HRQOL, and a systematic synthesis of population-based studies. Population-based samples provided a good basis for application of multivariable regression analyses, in which the effect of potential confounding variables (e.g., age, gender, BMI) can be accounted for.

The limitations are clarified as follows. The findings were primarily based on cross-sectional studies precluding a conclusion for the direction of the association between disordered eating and HRQOL. Although most primary studies have taken into account the effect of common confounding variables, such as sociodemographic variables and BMI, in multiple regression analyses, other studies have not, and some studies only used correlation analyses or simple independent sample tests (e.g., Mann-Whitney U tests). Finally, the number of studies in the meta-analysis was small due to methodological differences (e.g., assessment tools of the exposure and HRQOL) across studies; thus, the meta-analysis results should be interpreted with caution in terms of its generalizability to the broader population of children and adolescents.

## Conclusion

We have shown in the present systematic review that disordered eating is associated with decreased HRQOL in children and adolescents. More longitudinal and prospective studies are needed to ascertain the directionality in the effect of disordered eating on HRQOL among the population of children and adolescents. Future research is also needed to extend relevant studies among children and adolescents to facilitate quantitative synthesis of the association using meta-analysis. The findings of this review provide an evidence base that that might inform population-based health intervention strategies that promote healthy eating behaviors among children and adolescents in schools and communities. Preventing children and adolescents from developing disordered eating behaviors might help enhancing HRQOL in these individuals.

## Supporting information

S1 Table Literature search strategies for the databases of PUBMED, PSYCINFO and EMBASE. (DOC)

S2 Table PRISMA checklist. (DOC)
