# Supplementary material for: The association between disordered eating and health-related quality of life among children and adolescents: A systematic review of population-based studies
Source: PLoS One. 2019 Oct 4;14(10):e0222777. doi: 10.1371/journal.pone.0222777 (PMC6777752; doi:10.1371/journal.pone.0222777)
Supplement: S1 Table — (DOC) [file pone.0222777.s001.doc]

**S1 Table. Literature search strategy in electronic databases of PUBMED, PSYCINFO and EMBASE**

| **No** | **Searching terms** |
| --- | --- |
| **PUBMED** (1946 to July 18, 2018) | |
| 1 | dietary behav* OR eating behav* OR eating disorder* OR disordered eating OR binge eating OR anorexia nervosa OR bulimic eating disorder OR bulimia nervosa |
| 2 | health status OR quality of life |
| 3 | child* OR adolescen* OR teen* OR kids OR boys OR girls OR youth |
| 4 | 1 AND 2 AND 3 |
| 5 | Limit 4 to Humans and English |
| **Records retrieved: 1,950** | |
|  | |
| **PSYCINFO** (1980 to August 9, 2018) | |
| 1 | bulimia OR eating behavior OR eating disorders OR health behavior OR binge eating OR anorexia nervosa OR binge eating disorder |
| 2 | quality of life OR health |
| 3 | children OR adolescents OR adolescence OR childhood OR youth OR boys OR girls OR teen |
| 4 | 1 AND 2 AND 3 |
| 5 | limit 4 to (all journals and human AND english language AND peer reviewed journal AND yr='1980 -Current' |
| **Records retrieved: 1,102** | |
|  | |
|  | **EMBASE** (1966 to June 19, 2018) |
| 1 | health status OR health status indicator OR quality of life OR health related quality of life |
| 2 | child OR adolescent OR adolescence OR childhood OR youth OR boy OR girl |
| 3 | eating disorder OR eating behavior OR diet behavior OR dietary behavior OR disordered eating OR binge eating OR binge eating disorder OR anorexia nervosa OR bulimic eating disorder OR bulimia OR bulimia nervosa |
| 4 | 1 AND 2 AND 3 |
| **Records retrieved: 691** | |
| **Total records retrieved: 3,743** | |

* exploded
